# The PrEP You Want: A Web-Based Survey of Online Cross-Border Shopping for HIV Prophylaxis Medications

**DOI:** 10.2196/12076

**Published:** 2019-07-22

**Authors:** Ben Walmsley, Dan Gallant, Mark Naccarato, Mark Hull, Alex Smith, Darrell Hoi-San Tan

**Affiliations:** 1 Division of Infectious Diseases St Michael's Hospital Toronto, ON Canada; 2 Gay Men’s Sexual Health Alliance Toronto, ON Canada; 3 Division of Infectious Diseases University of British Columbia Vancouver, BC Canada; 4 Cumming School of Medicine University of Calgary Calgary, AB Canada

**Keywords:** pre-exposure prophylaxis, tenofovir disoproxil fumarate/emtricitabine, generic antiretroviral drugs, online medication shopping, men who have sex with men, HIV

## Abstract

**Background:**

In response to the high cost of HIV pre-exposure prophylaxis (PrEP) medications in Canada, community organizations have created internet-based guides detailing how to legally order generic medications online and travel to collect them in the United States. However, little is known about the patients following these guides.

**Objective:**

Our primary objective was to measure the proportion of Ontario gay, bisexual, and other men who have sex with men (GBMSM) accessing these online guides who intended to use the border-crossing approach. Our secondary objectives were to explore their demographic characteristics, their completion of the steps in the border-crossing approach, and the barriers they perceived.

**Methods:**

Between July 20, 2017, and May 18, 2018, we administered two online surveys of GBMSM accessing an online border-crossing guide posted by a gay men’s health organization in Ontario. Participants completed an open baseline survey posted on the border-crossing guide’s Web page and a follow-up survey 3 months later. The data were analyzed using descriptive statistics. We used multivariable logistic regression to identify characteristics associated with the intention to use the border-crossing approach.

**Results:**

Most of the 141 participants were young (median age 23, interquartile range 22-25 years) and black (79.4%; 112/141) GBMSM who had completed a college or an undergraduate degree (62.4%; 88/141). In addition, 19.9% (28/141) of them reported a total family income less than Can $30,000 and another 53.9% (76/141) reported income between Can $30,000 and Can $60,000. 54.6% (76/141) paid for medications entirely out of pocket. Most participants indicated that they were likely to complete a border-crossing approach: 80.1% (113/141) at baseline and 79.1% (87/110) at follow-up. The characteristics associated with the intention to use the approach included being black (adjusted odds ratio [aOR] 5.73, 95% CI 2.06-16.61), paying for medications out of pocket (aOR 5.18, 95% CI 1.82-17.04), and having a provider who was thought to be willing to prescribe PrEP (aOR 4.42, 95% CI 1.63-12.41). Comparing baseline and follow-up for the 110 participants who completed both surveys, 65.4% (72/110) and 80.0% (88/110) had discussed PrEP with a health care provider, 18.1% (20/110) and 25.4% (28/110) had obtained a PrEP prescription, and 8.2% (9/110) and 5.5% (6/110) had ordered medications to that mailbox, whereas only 1.0% (1/110) and 0.0% (0/110) had crossed the border to collect them at baseline and follow-up, respectively. Reported barriers included perceived concerns about the approach’s legality (56.0%; 79/141), the security of personal health information (39.0%; 55/141), and the safety of online vendors (38.3%; 54/141).

**Conclusions:**

Despite high interest in pursuing an online border-crossing approach to get PrEP medications, such an approach may not be a viable option for PrEP scale-up among interested GBMSM because of logistical challenges and perceptions of safety and legitimacy.

## Introduction

### Background

HIV infection rates remain high among gay, bisexual, and other men who have sex with men (GBMSM) living in Canada, accounting for an estimated 47.9% of the nation’s 2328 new adult cases in 2016 [1]. Pre-exposure prophylaxis (PrEP) is a promising strategy for preventing HIV infection [2]. Pharmacokinetic models suggest that consistent use of PrEP, which currently involves regular oral dosing of two coformulated antiretroviral medications, tenofovir disoproxil fumarate and emtricitabine (TDF/FTC), reduces the risk of HIV infection by almost 100% in GBMSM [3-5]. Furthermore, we have documented high and increasing willingness to use PrEP among GBMSM living in Canada. Although 33.3% of a sample of GBMSM attending a major Toronto sexual health clinic for HIV testing in 2011 said they would definitely be willing to use PrEP, this proportion increased to 52.5% in 2015 [6].

Despite this interest, the cost of PrEP medications has been a barrier to its uptake in Canada, where health services are publicly funded but medication costs are not universally covered. Brand name TDF/FTC (Truvada) costs approximately Can $876 per month in Ontario [7], and although generic TDF/FTC is less expensive at approximately Can $220 per month, this cost remains excessive for many. PrEP is publicly subsidized in some but not all Canadian jurisdictions at present, and in some cases, including Ontario, coverage may be only partial, requiring out-of-pocket copayments. Accordingly, the cost of PrEP is still prohibitive for many Canadians [8]. PrEP access in other high-income jurisdictions is variable, although notable examples of truly universal PrEP coverage have begun to emerge, including in France, Scotland, and New South Wales.

In response to this barrier in Canada, motivated patients have been pursuing alternate strategies to obtain less expensive generic PrEP medication, including crossing the border to the United States to pick up PrEP that they have ordered online [9]. Although having prescription drugs shipped directly into Canada for personal use is illegal [10], individuals crossing the border can legally import up to 90 days’ worth of medication for personal use. Therefore, innovative activists and community organizations have created online resources detailing how a border-crossing approach can be used to legally acquire more affordable PrEP. The process involves four basic steps: (1) getting a prescription, (2) getting a mailbox in a US border location, (3) ordering medication online that ships to that location, and (4) picking up medication from that US location and crossing the border back to Canada every 3 months as needed. This approach is legal and, thus, its safety should be similar to crossing the border for any other reason. Reports from community members and clinicians alike indicate that this approach has been widely used in British Columbia [9,11], and a new campaign began promoting the strategy in Ontario in mid-2017. However, there are few data on the characteristics and motivations of the strategy’s users in any setting and any perceived or actual barriers they may face.

### Objectives

To address these gaps, we conducted two Web-based surveys, one baseline and one follow-up, of GBMSM who were considering following the border-crossing approach as described by the major Ontario community organization championing the new campaign, the Gay Men’s Sexual Health Alliance (GMSH), in an online resource titled *The PrEP You Want* [12]. Our primary objective was to measure the proportion of individuals accessing the resource who indicated an intention to use the border-crossing approach. Our secondary objectives were to explore their characteristics, their completion of the steps in the border-crossing process (Textbox 1), and the barriers they perceived.

**Steps in a cascade of online shopping and border crossing to obtain pre-exposure prophylaxis (PrEP).**

1. Has decided to get PrEP
2. Has discussed PrEP with a health care provider
3. Has found a health care provider who will prescribe PrEP
4. Has decided to obtain PrEP with the border-crossing strategy
5. Has obtained a prescription from a health care provider
6. Has secured a mailbox in a US city
7. Has ordered medication online for shipment to that US mailbox
8. Has picked up PrEP medication from the US mailbox and crossed the border back to Canada

## Methods

### Study Design

Between July 20, 2017, and May 18, 2018, we administered two online surveys, one baseline and one follow-up, of GBMSM considering the border-crossing approach. The study was an open survey: to enter the study, anyone who self-reported that they met the eligibility criteria could access it by clicking on an online study link. We posted the link on the GMSH online information page, and the GMSH advertised this information page on several websites (eg, AIDS Committee of Toronto website, *PrEP—Canada: Rethinking HIV Prevention* Facebook page). We sent all the eligible participants a baseline questionnaire along with a unique participant study code by email, and after 3 months, we sent a follow-up questionnaire to capture updated information on their experience. For both the baseline and the follow-up surveys, we contacted participants who had not yet completed their questionnaires two more times with reminders.

### Participants

The participants were eligible if they self-identified as being gay, bisexual, or otherwise a man who has sex with men; were residents of Ontario; were first-time participants in the study; and were able to read and write English. In addition, they had to report having read the GMSH online information page and having determined whether they were likely to use the strategy or not. All participants were offered a Can $5 electronic gift card on completion of the baseline questionnaire, and a Can $10 electronic gift card on completion of the follow-up questionnaire.

### Survey Instrument

We designed both the 37-item baseline questionnaire and the 27-item follow-up questionnaire based on previous surveys of potential PrEP users [13] and the language of the GMSH online resource. The questionnaires covered the following domains: demographics; HIV risk behaviors; health care access; knowledge and experience of PrEP; and interest and experience with the border-crossing strategy. We developed the demographic section to match a standardized health equity questionnaire developed by major health care organizations in Toronto [14]. The HIV risk section included all items from the High Incidence Risk Index for men who have sex with men (HIRI-MSM), a HIV risk index developed and validated by the US Centers for Disease Control for its use in predicting seroconversion in GBMSM [15]. It should be noted that although the HIRI-MSM questions are based on 6-month look-back intervals, our questionnaires used 3-month intervals to avoid overlap between the baseline and follow-up responses; these 3-month responses were then doubled to estimate the final HIRI-MSM scores. We housed both surveys on the website *HostedinCanadaSurveys*.

### Analysis

Data were primarily analyzed with descriptive statistics, and the analysis was conducted separately for the baseline data and the follow-up data. For the primary objective, we calculated the proportion of individuals who answered *likely* or *very likely* to the question: “How likely is it that you will use this ‘The PrEP You Want: How to Order PrEP Online’ approach to buy generic PrEP drugs?”. For the secondary objectives, we calculated the proportion of individuals who reported completing each of the cascade of steps in Textbox 1 and the proportion reporting a variety of barriers to carrying out *The PrEP You Want* approach.

As an exploratory analysis, we constructed univariable and multivariable logistic regression models quantifying the relationship between respondent characteristics and interest in using the border-crossing approach, first using the baseline data only and subsequently using the follow-up data. When building the multivariable models, we first excluded variables that were highly correlated and then included variables in the final model if they modified the beta estimate for the primary predictor variable of interest, medication insurance status, by more than 10%. To investigate the possibility of falsified responses, we conducted a sensitivity analysis comparing the demographic characteristics and primary outcome responses among the decile of respondents with the shortest survey completion times with those of the total sample. In addition, we conducted a log file analysis to confirm that responses corresponded with unique internet protocol addresses. All quantitative analysis was done using R software (R Core Team).

### Sample Size Considerations

The sample size for this study was based on the minimum required sample size to ascertain, among individuals accessing the website, the proportion indicating an intention to use the border-crossing strategy with reasonable precision. There were no previous data to our knowledge on the likely value of this proportion, so we conservatively assumed that the true proportion was 0.5 (the value that generates the largest potential sample size requirements). We determined that a target sample size of 150 respondents for the baseline questionnaire would allow us to estimate the true prevalence with reasonable precision (plus or minus 8%) [16].

### Ethical Approval

This study was approved by the University of Toronto’s HIV Research Ethics Board and the St Michael’s Hospital Research Ethics Board before any study activities were initiated. All the participants documented their consent online before beginning either of the surveys.

## Results

### Survey Response

All 163 individuals who provided a valid email address were sent the baseline survey. Of the 158 participants who then began the baseline survey, 17 were excluded because of incomplete responses (n=5), repeat responses (n=6), and being non-Ontarian (n=6). Of the 141 participants included in the final baseline analysis, 110 provided complete responses to the follow-up survey and were included in the final follow-up analysis. The number of unique site visitors (ie, participants exposed to the survey link) could not be calculated as the survey link was likely shared through multiple informal channels.

### Demographics

Participant characteristics are described in Table 1. Median age was 23 (interquartile range [IQR] 22-25) years at baseline, and none of the respondents were below 18 years old. Most of the baseline participants were black (79.4%; 112/141), gay (87.2%; 123/141), and male (98.6%; 139/141), and 62.4% (88/141) had a college or undergraduate education or higher. Less than half (45.4%; 64/141) had drug coverage but most (83.0%; 117/141) had a family doctor or nurse practitioner with whom they felt comfortable discussing their sexual health. An HIRI-MSM score could be calculated for the 139 participants who answered all the requisite questions. The median HIRI-MSM score was 29 (IQR 26-30), and 97.8% (136/139) of men scored more than or equal to 10, meeting the index’s criteria for high HIV risk. Characteristics of the 110 participants who completed the follow-up survey and the 31 who were lost to follow-up were similar (data not shown). None of the demographic or behavioral variables were significantly associated with completion of the follow-up survey.

**Table 1:** Participant characteristics.

Characteristic		Baseline (n=141)	Follow-up (n=110)
Age (years), median (IQR^a^)		23 (22-25)	23 (22-25)
**Ethnicity, n (%)**			
	Black	112 (79)	88 (80)
	White	16 (11)	13 (12)
	Southeast Asian	5 (4)	4 (4)
	Latin American	3 (2)	2 (2)
	East Asian	3 (2)	1 (1)
	South Asian	2 (1)	2 (2)
**Education, n (%)**			
	High school diploma or less	53 (38)	38 (35)
	College/undergraduate degree	81 (57)	65 (59)
	Professional or graduate degree	7 (5)	7 (6)
**Total family income (Can $^b^), n (%)**			
	0-29,999	28 (20)	18 (16)
	30,000-59,000	76 (54)	64 (58)
	>60,000	35 (25)	26 (24)
**Medication payment^c^, n (%)**			
	Private insurance	56 (40)	43 (39)
	Out of pocket	77 (55)	63 (57)
	Government drug benefit	8 (6)	4 (4)
Has a primary care provider with whom they feel comfortable discussing sexual health, n (%)		117 (83)	103 (94)
Has previously used pre-exposure prophylaxis, n (%)		37 (26)	35 (32)
**Drug use in the past 6 months, n** **(%)**			
	Alcohol	116 (82)	105 (95)
	Marijuana (weed)	47 (33)	42 (38)
	Cocaine	23 (16)	24 (22)
	Poppers (amyl nitrate)	22 (16)	12 (11)
	Methamphetamines (crystal and speed)	9 (6)	4 (4)
	Injectable drugs	3 (2)	2 (2)
**Lifetime diagnosis of a sexually transmitted infection, n** **(%)**			
	Genital herpes	23 (16)	10 (9)
	Gonorrhea	12 (9)	7 (6)
	Chlamydia	8 (6)	3 (3)
	Genital or anal warts	6 (4)	3 (3)
	Syphilis	3 (2)	4 (4)
Ever participated in chemsex/party and play^d^, n (%)		50 (35)	43 (39)
High Incidence Risk Index for men who have sex with men, median (IQR)^e^		29 (26-30)	29 (26-32)
Number of male sex partners in past 3 months, median (IQR)		5 (4-7)	5 (3-7)
Number of times having condomless receptive anal sex in past 3 months, median (IQR)		6 (3-14)	8 (5-11)
Number of partners known to be HIV positive in past 3 months, median (IQR)		0 (0-1)	0 (0-1)

^a^IQR: interquartile range.

^b^Two participants responded *Don’t know* in both baseline and follow-up.

^c^Two participants did not respond in the baseline.

^d^One participant did not respond in the baseline.

^e^Two participants did not answer all of the requisite risk questions in the baseline survey.

### Primary Outcomes

In the primary analysis, 80.1% of participants indicated that they were likely to use the border-crossing approach. This proportion was similar at follow-up (79.1%; 87/110). More than half of the participants had first heard about the approach from a friend (55.3%; 78/141), but participant responses varied by ethnicity: the leading response among black participants was *from a friend* (66.1%; 74/112), compared with only 20.6%; 6/29) of nonblack respondents, and the leading response among nonblack participants was *media or online* (48.5%, 14/29).

**Figure 1:**
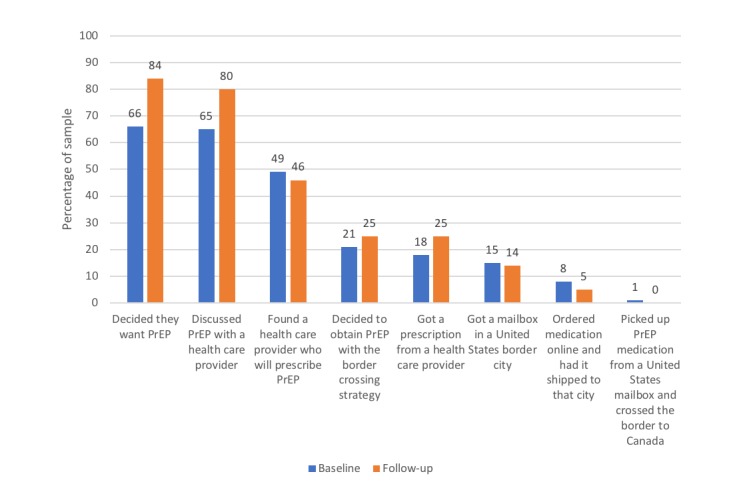
Steps completed in the border crossing for pre-exposure prophylaxis cascade at baseline and 3-months follow-up (n=110 participants who completed both surveys). PrEP: pre-exposure prophylaxis.

At baseline, more than half of the participants had both decided that they wanted PrEP (67.4%, 95/141) and already discussed PrEP with a health care provider (62.4%, 88/141). However, only 46.1% (65/141) had found a health care provider who would be willing to prescribe PrEP and only 24.1% (34/141) had already obtained a prescription. Few participants had completed the steps of the cascade specific to border crossing such as obtaining a mailbox in the United States (Figure 1). At the 3-month follow-up, the 110 participants who had completed both surveys showed increased completion of preliminary steps but decreased completion of the final steps related to border crossing (Figure 1). Notably, only 10% (11/110) of follow-up respondents had decided to obtain PrEP with a different method such as purchasing from a Canadian pharmacy.

We explored the variables associated with our primary outcome (likelihood of using the border-crossing approach) in exploratory logistic regression analyses separately for the baseline and follow-up data (Table 2). At baseline, factors associated with likeliness to use the border-crossing approach in both univariable and multivariable models included being black (adjusted odds ratio [aOR] 5.73, 95% CI 2.06-16.61), paying for medications out of pocket (aOR 5.18, 95% CI 1.82-17.04), and having a provider who was thought to be willing to prescribe PrEP (aOR 4.42, 95% CI 1.63-12.41). At follow-up, the findings were generally similar, as shown in Table 2. Only 9 individuals changed from being likely to unlikely to use the border-crossing approach between baseline and follow-up, whereas 8 did the reverse.

**Table 2:** Association between participant characteristics and the likelihood of using the border-crossing approach.

Participant characteristic			Baseline				Follow-up			
			Univariable		Multivariable		Univariable		Multivariable	
			OR^a^ (95% CI)	*P* value	OR (95% CI)	*P* value	OR (95% CI)	*P* value	OR (95% CI)	*P* value
**Demographics**										
	Age (years)		0.74 (0.61-0.87)	.001	—^b^	—	0.86 (0.73-0.97)	.03	—	—
	**Ethnicity (ref: nonblack^c^)**									
		Black	8.20 (3.30-21.19)	<.001	5.73 (2.06-16.61)	.002	15.36	<.001	14.42 (4.48-53.11)	<.001
	**Education (ref: high school diploma or less)**									
		College/undergraduate degree or more	0.49 (0.18-1.19)	.10	—	—	0.79 (0.28-2.07)	.64	—	—
	**Total family income (Can $; ref: 0-29,999)**									
		30,000-59,000	1.80 (0.60-5.14)	.29	—	—	3.49 (0.99-12.21)	.05	—	—
		>60,000	0.76 (0.24-2.31)	.62	—	—	0.69 (0.19-2.34)	.69	—	—
**PrEP^d^ access**										
	**Medication insurance (ref: private insurance or full government coverage)**									
		Out of pocket	5.75 (2.26-16.72)	<.001	5.18 (1.82-17.04)	.003	7.20 (2.59-23.60)	<.001	6.69 (2.07-25.91)	.003
	Has a primary care provider that the respondent thinks would be willing to prescribe PrEP		6.39 (2.66-16.00)	<.001	4.42 (1.63-12.41)	.004	5.77 (2.18-15.91)	<.001	—	—
	Has previously used PrEP		5.83 (1.62-37.45)	.02	—	—	3.88 (1.21-17.37)	.04	—	—
	Able and willing to pay more than Can $100 per month for PrEP		0.66 (0.28-1.53)	.31	—	—	1.02 (0.37-3.10)	.97	—	—
**Risk factors**										
	High Incidence Risk Index for men who have sex with men score		1.09 (1.03-1.15)	.002	—	—	1.10 (1.04-1.18)	.002	—	—
	Number of male sex partners in the past 3 months		1.05 (0.90-1.23)	.63	—	—	1.03 (0.87-1.23)	.76	—	—
	**Perceived chance of getting HIV in the next year (ref: little to none)**									
		More than a little or greater	0.97 (0.37-2.87)	.91	—	—	0.53 (0.20-1.42)	.19	—	—
		Ever diagnosed with a sexually transmitted infection	1.94 (0.72-6.18)	.19	—	—	2.38 (0.80-8.76)	.15	—	—
^ ^	^ ^	Party drug use in the past 6 months^e^	1.59 (0.67-4.13)	.31	—	—	1.61 (0.62-4.56)	.34	—	—
	Ever participated in chemsex/party and play		2.34 (0.93-6.76)	.12	—	—	—	.16		—
**Perceptions of the border-crossing approach**										
	**First heard about the approach from (ref: friend[s])**									
		Other (health care provider, community organization, online, etc)	0.25 (0.10-0.60)	.003	—	—	0.20 (0.06-0.52)	.002	—	—
		Number of barriers perceived/anticipated	1.19 (0.88-1.65)	.28	—	—	0.97 (0.73-1.31)	.87	—	—
		Concerned about the legality of the approach	1.47 (0.63-3.44)	.40	—	—	1.67 (0.66-4.39)	.28	—	—
		Knows someone who uses the approach	6.70 (2.53-21.20)	<.001	—	—	2.42 (0.95-6.97)	.07	—	—

^a^OR: odds ratio.

^b^Further analysis not run on these variables.

^c^White, East Asian, South Asian, and Latin American.

^d^PrEP: pre-exposure prophylaxis.

^e^Party drugs specified as methamphetamines, gamma-hydroxybutyrate, amyl nitrate, cocaine, crack, ketamine, and injectable drugs.

### Motivators and Barriers

The most commonly identified motivator to using the border-crossing approach was the high cost of PrEP at Canadian pharmacies (59.6%, 84/141), with 57.4% (81/141)of the participants identifying a maximum monthly amount that they would be able and willing to pay for PrEP that was less than Can $100. The second most commonly identified motivator was already knowing someone who uses the approach (52.5%, 74/141).

**Figure 2:**
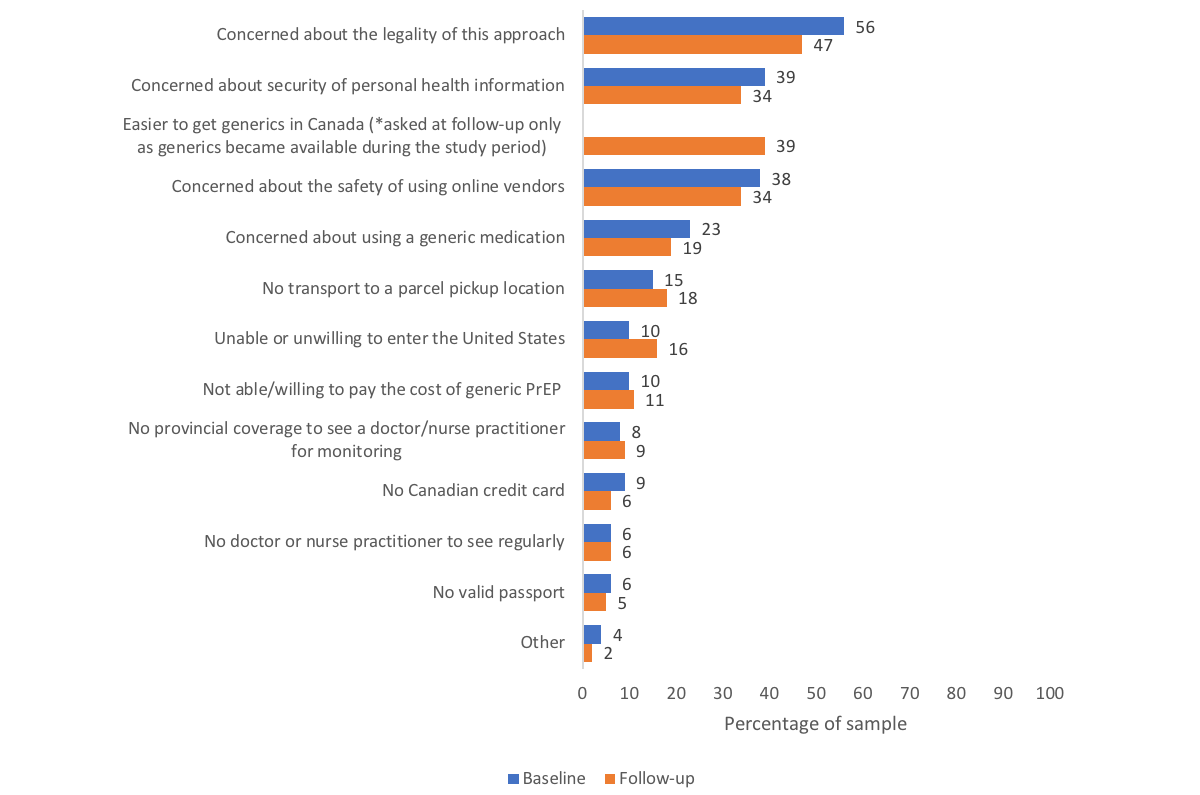
Reported barriers to using the border-crossing strategy. PrEP: pre-exposure prophylaxis.

The most commonly identified barriers to using the border-crossing approach at baseline were concerns about the legality of the approach (56.0%, 79/141), the safety of using online vendors (38.3%, 54/141), and the security of personal health information (36.9%; 52/141; Figure 2). The findings for motivators and barriers were similar at follow-up, except that 40.9% (45/110) of participants identified a new barrier being that it had become easier to get generic PrEP in Canada instead. We added this response option for the follow-up questionnaire only because domestically manufactured generic PrEP became available and partially publicly subsidized in Ontario in late 2017, before follow-up data collection began. Although 44.5% (49/110) of follow-up participants reported that they would be less likely to use the border-crossing approach given these policy changes, 86.3% (95/110) were unaware of the new changes before beginning the follow-up survey.

### Checking Response Quality

We conducted a sensitivity analysis comparing the decile of respondents (n=14) with the shortest survey completion times to the total sample. Response proportions for both groups were consistent across both the primary outcome and the demographic characteristics included in Table 1 (data not shown).

## Discussion

### Principal Findings

Understanding both who is considering online alternatives for PrEP access and why they are doing so is vital to understanding gaps in PrEP implementation. In our sample of mostly Toronto-based, black GBMSM, we found high interest in pursuing an innovative approach to obtaining PrEP medications, involving online ordering and cross-border retrieval, measuring 80.1% (113/141) at baseline and 79.1% (87/110) at follow-up. Participants identified both key motivators (eg, knowing someone who uses a border-crossing approach and high domestic costs) and barriers (eg, concerns about the legality of such an approach, security of personal health information, and the safety of online vendors) to pursuing this strategy. To our knowledge, this is the first study regarding this Web-based, border-crossing approach to obtain PrEP medications.

The proportion of black participants was surprisingly high at 79.0% (112/141) , given that less than 5% of Ontario’s men identify as black [17], raising the possibility of sampling bias. However, the direction of any such bias is unclear, and our primary purpose was to assess the intentions of those accessing the online resource rather than those of the GBMSM population as a whole. We suspect that the high number of black participants is a result of social networks circulating the online link to the study survey; 66.1% (74/112) of black participants reported that they had heard of the border-crossing strategy from friends vs only 22.9% (6/29) of nonblack participants. Given that North American black GBMSM have historically been both socioeconomically disadvantaged and harder to engage in HIV prevention research [18-21], our high number of black respondents may reflect an unmet need for alternative methods of PrEP access in this population, and the advantages of using Web-based approaches for reaching and studying the needs of marginalized populations.

### Comparison With Previous Work

Online PrEP shopping has been a major alternative approach for accessing PrEP among GBMSM in other jurisdictions such as the United Kingdom and Australia. Online purchasing of inexpensive PrEP likely played a causal role in lowering the rates of HIV infection among GBMSM at sexual health clinics in the United Kingdom [22,23] and filled a critical access gap in the formal health care system, where the National Health Service has decided not to fund PrEP medication [24]. UK online information pages such as *I want PrEP Now* were precursors to the Web pages created by Canadian activists and community organizations (eg, *The Davie Buyers Club* and the GMSH’s *The PrEP You Want* campaign) [25], and many of these pages refer users to the same pool of Web-based international generic medication suppliers. Similarly in Australia, online purchasing, often championed by community groups and websites, was the primary means of PrEP access before the eventual public listing of PrEP on the national drug benefit scheme in 2018 [26,27]. In British Columbia, limited data from 2017 suggest that 200 individuals accessed PrEP through *The Davie Buyers Club* over a 3-month period, though detailed information on the characteristics and experiences of these individuals is lacking [9]. Although there has been some concern over the quality of medications ordered online, both in our sample and in the literature [28,29], evidence from both the UK and Canadian contexts suggests that online PrEP distributors provide safe and effective generic medications [30,31]. However, given the complexity and inconvenience involved in border crossing to obtain PrEP, we contend that this strategy is not the optimal way to make this primary prevention strategy available in Canada, and PrEP needs to be more widely available to Canadians at risk.

Following in the mold of HIV cascades, hypothetical PrEP cascades have been proposed and used to identify bottlenecks at different stages of PrEP implementation [32-34]. For instance, our previous hypothetical PrEP cascade found that although 64.4% of Toronto GBMSM undergoing anonymous HIV testing were at objectively high HIV risk, 68.3% did not perceive themselves to be at elevated risk and 47.6% lacked access to a family doctor [7]. Our hypothetical cascade of border-crossing steps identified parallel bottlenecks in accessing health care providers and obtaining PrEP prescriptions. These data highlight the need for continued efforts to make PrEP providers and PrEP medication widely accessible, even in large urban centers such as Toronto.

Two positive developments in Ontario’s PrEP access landscape have been the entry of generics into the Canadian PrEP market (August 2017) and the government’s initiation of partial public subsidization (September 2017). As a result, PrEP costs have been reduced (the generic currently costs approximately Can $220 per month) or largely covered (for Ontarians meeting the criteria for public drug benefits, sometimes involving copayment) [35]. As indicated by the 40.9% (45/110) of follow-up study participants who cited the availability of cheap generics in Canada as a deterrent to pursuing a border-crossing approach, this shift in cost has made conventional approaches to obtaining PrEP more viable for many potential users. However, despite the availability of reduced-cost generics in Ontario, our previous work demonstrated that PrEP could in some cases be obtained online for as little as Can $33 per month [31], representing a cost savings of approximately $187 per month over the generic TDF/FTC available in Ontario.

However, PrEP remains financially inaccessible for many potential users in Canada. This held true in our sample where 57.4% (81/141) participants identified a maximum monthly amount that they would be able and willing to pay for PrEP that was less than Can $100. Although Can $33 per month for online PrEP is within this range, the attrition we observed in our border-crossing cascade suggests that relying on online purchasing, even at this price, is not a viable public health strategy for improving PrEP accessibility. Given the recent findings of real-world reductions in HIV incidence of up to 100% in PrEP users, and PrEP’s role in the substantial reductions in HIV incidence in settings such as San Francisco and New South Wales [36-39], Canada’s inconsistent access to this major public health tool emphasizes the urgent need for truly universal coverage. This is particularly important given the failure of HIV incidence to decline among Canadian GBMSM in recent years [40].

### Limitations

This study has several limitations that warrant consideration. First, because our study was an anonymous open online survey, it was not possible to exclude repeat participants, and some may have been motivated by the modest financial compensation. We attempted to minimize repeats by requiring participants to enter unique study codes that were assigned to each respondent’s email address. We also checked for possible falsified responses by conducting a sensitivity analysis, which showed consistency between the demographic characteristics and primary outcome responses among the fastest decile of responders and the total sample. Second, hypothetical bias may have inflated the number of participants indicating likeliness to use this fairly time-intensive border-crossing approach [41]. We addressed this possibility in part by asking about the actual completion of cascade steps at 3-month follow-up. In addition, however, the relatively short time frame (3 months) between baseline and follow-up may still have been insufficient to capture participants’ progress in using the border-crossing approach. These last two biases may in part explain why we found no participants who had completed every step of the border-crossing approach at follow-up. Furthermore, it is notable that although half of the respondents reportedly knew someone using the border-crossing approach, very few respondents actually did so themselves. This discrepancy raises the possibilities that respondents were underreporting their use of the procedure, that our study underrecruited individuals already successfully using this strategy, or that a limited number of border crossers were known to a large number of respondents. Finally, because we calculated HIRI-MSM scores for our participants by doubling the responses given for the past 3 months (rather than directly asking about the past 6 months), they may have deviated slightly from *true* scores. Moreover, HIRI-MSM scoring may be less relevant to the contemporary context as HIV risk through condomless sex can be reduced to zero with HIV treatment as prevention and/or PrEP. Regardless, our sample was likely still at high HIV risk because of its young age, high mean number of sex partners, frequent condomless receptive anal sex, and a 35% participation rate in chemsex.

### Conclusions

PrEP is a promising tool for curbing high rates of HIV in GBMSM communities, but access remains highly variable in Canada. Our results suggest that despite the falling price of PrEP medications and some government subsidization, many potential PrEP users remain interested in alternative, cheaper methods of obtaining PrEP medications such as online shopping and border crossing. Effective PrEP implementation will require alternative strategies such as universal public coverage to ensure readily accessible PrEP medications for GBMSM living in Canada.

## Abbreviations

aOR: adjusted odds ratio
GBMSM: gay, bisexual, and other men who have sex with men
GMSH: Gay Men’s Sexual Health Alliance
HIRI-MSM: High Incidence Risk Index for men who have sex with men
IQR: interquartile range
PrEP: pre-exposure prophylaxis
TDF/FTC: tenofovir disoproxil fumarate and emtricitabine
